# Transcriptomic analysis reveals potential genes involved in tanshinone biosynthesis in *Salvia miltiorrhiza*

**DOI:** 10.1038/s41598-019-51535-9

**Published:** 2019-10-17

**Authors:** Yujie Chang, Meizhen Wang, Jiang Li, Shanfa Lu

**Affiliations:** 1 Institute of Medicinal Plant Development, Chinese Academy of Medical Sciences & Peking Union Medical College, Beijing, 100193 China; 2 Institute of Crop Sciences, Chinese Academy of Agricultural Sciences, Beijing, 100081 China; 3 Beijing Advanced Innovation Center for Tree Breeding by Molecular Design, Beijing Forestry University, Beijing, 100083 China

**Keywords:** Transcriptomics, Secondary metabolism

## Abstract

Tanshinones are important bioactive components in *Salvia miltiorrhiza* and mainly accumulate in the periderms of mature roots. Tanshinone biosynthesis is a complicated process, and little is known about the third stage of the pathway. To investigate potential genes that are responsible for tanshinone biosynthesis, we conducted transcriptome profiling analysis of two *S. miltiorrhiza* cultivars. Differential expression analysis provided 2,149 differentially expressed genes (DEGs) for further analysis. GO and KEGG analysis showed that the DEGs were mainly associated with the biosynthesis of secondary metabolites. Weighted gene coexpression network analysis (WGCNA) was further performed to identify a “cyan” module associated with tanshinone biosynthesis. In this module, 25 cytochromes P450 (CYPs), three 2-oxoglutarate-dependent dioxygenases (2OGDs), one short-chain alcohol dehydrogenases (SDRs) and eight transcription factors were found to be likely involved in tanshinone biosynthesis. Among these CYPs, 14 CYPs have been reported previously, and 11 CYPs were identified in this study. Expression analysis showed that four newly identified CYPs were upregulated upon application of MeJA, suggesting their possible roles in tanshinone biosynthesis. Overall, this study not only identified candidate genes involved in tanshinone biosynthesis but also provided a basis for characterization of genes involved in important active ingredients of other traditional Chinese medicinal plants.

## Introduction

*Salvia miltiorrhiza* (typically called danshen in Chinese) belongs to the *Lamiaceae* family and is a commonly used traditional Chinese medicine with great economic and medicinal value. The *S. miltiorrhiza* root has been widely used for the treatment of cardiovascular and cerebrovascular diseases^1^. Lipid-soluble tanshinones and water-soluble phenolic compounds are the two major groups of active components in *S. miltiorrhiza*^2^.

The red color of *S. miltiorrhiza* roots is largely ascribed to tanshinones and related quinones^3^. Tanshinones are abietane-type diterpenoids. They mainly accumulate in the periderms of mature roots^4^. To date, more than 40 tanshinones, including tanshinone I, tanshinone IIA, tanshinone IIB, cryptotanshinone, and dihydrotanshinone, have been isolated and structurally characterized^2^. Due to the medicinal and economic value of danshen, the production of tanshinone content can be effectively enhanced through genetic engineering or synthetic biology approaches. Therefore, elucidating the biosynthesis of tanshinones and identifying the key enzyme genes in the biosynthetic pathway are necessary for improving the production of tanshinones.

**Figure 1:**
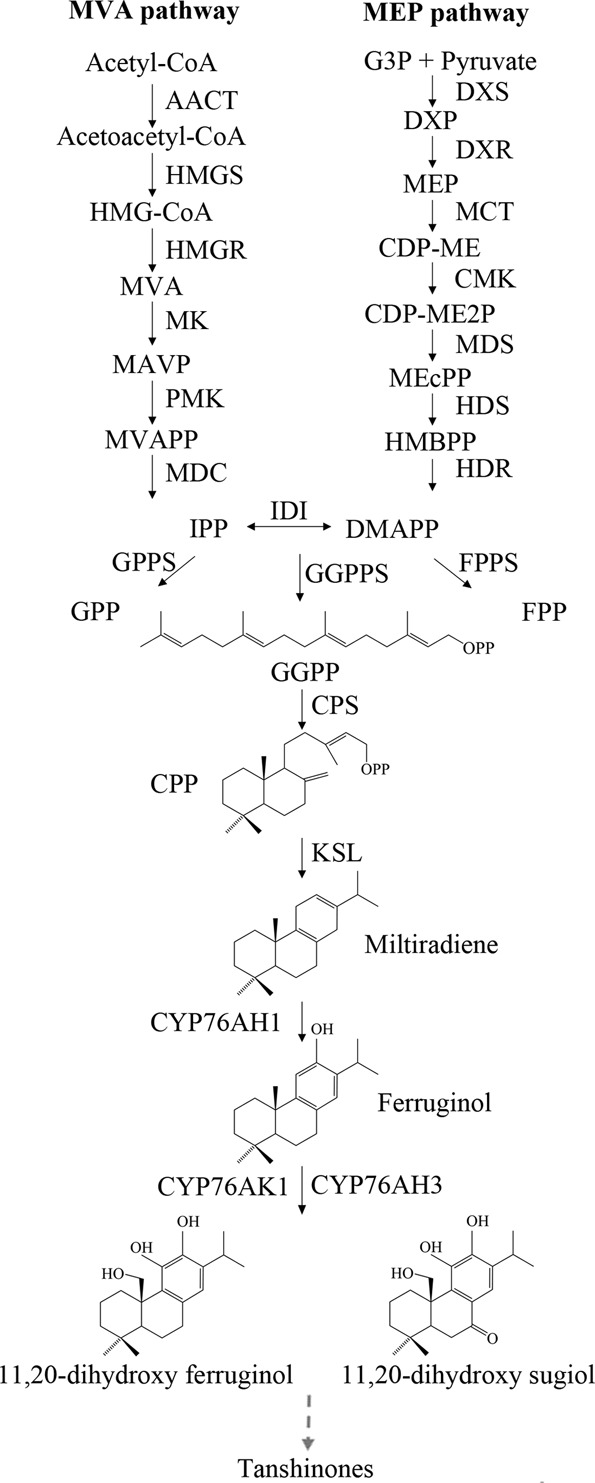
A schematic of the tanshinone biosynthetic pathway. AACT: acetyl-CoA C-acetyltransferase, HMGS: hydroxymethylglutaryl-CoA synthase, HMGR: 3-hydroxy-3-methylglutaryl-CoA reductase, MK: mevalonate kinase, PMK: 5-phosphomevalonate kinase, MDC: mevalonate pyrophosphate decarboxylase, DXS: 1-deoxy-D-xylulose-5-phosphate synthase, DXR: 1-deoxy-D-xylulose 5-phosphate reductoisomerase, MCT: 2-C-methyl-D-erythritol 4-phosphate cytidylyltransferase, CMK: 4-diphosphocytidyl-2-C-methyl-D-erythritol kinase, MDS: 2-C-methyl-D-erythritol 2,4-cyclodiphosphate synthase, HDS: 4-hydroxy-3-methylbut-2-enyl diphosphate synthase, HDR: 4-hydroxy-3-methylbut-2-enyl diphosphate reductase, IPP: isopentenyl pyrophosphate, DMAPP: dimethylallyl pyrophosphate, IDI: isopentenyl pyrophosphate isomerase, GGPP: geranylgeranyl diphosphate, GGPPS: geranylgeranyl diphosphate synthase, CPP: copalyl diphosphate, CPS: CPP synthase, and KSL: kaurene synthase-like cyclase.

The putative biosynthetic pathway of tanshinones can be divided into three stages (Fig. 1). The first stage is the formation of terpenoid precursors. Isopentenyl diphosphate (IPP) and its isomer dimethylallyl diphosphate (DMAPP) are produced through the following two biosynthetic pathways: the mevalonate (MVA) pathway and the methylerythritol phosphate (MEP) pathway. The second stage is the construction of tanshinone skeletons. IPP and DMAPP are further catalyzed by geranylgeranyl diphosphate synthase (GGPPS) to form geranylgeranyl diphosphate (GGPP)^5^. The third stage is the formation of diverse tanshinones. Currently, little is known about the third stage of tanshinone biosynthesis. In the third stage of tanshinone biosynthesis, tanshinone skeletons produce various tanshinones by the catalysis of terpene synthases and by the modification of terpenoid-modifying enzymes^2,6^. Copalyl diphosphate synthase (CPS) and kaurene synthase-like cyclase (KSL) are two key terpenoid synthases involved in the biosynthesis of tanshinones. Studies have reported that SmCPS1 and SmCPS2 react with GGPP to form normal copalyl diphosphate (CPP) and that SmKSL1 further catalyzes the production of miltiradiene^7,8^. Twelve *SmCPS* and nine *SmKSL* homologs have been previously identified in *S. miltiorrhiza*^4^. Miltiradiene is the precursor of tanshinone and is further modified by cytochrome P450s (CYPs), dehydrogenases, demethylases, and other enzymes to produce structurally different compounds^3^. SmCYP76AH1 has been suggested to catalyze miltiradiene to produce ferruginol *in vitro* and *in vivo*^9^. RNA interference of *SmCYP76AH1* in hairy roots led to significantly increased accumulation of miltiradiene, and the concentration of ferruginol decreased, revealing the key role in the biosynthesis of tanshinones^10^. Coexpression analysis showed that *SmCYP76AH3* and *SmCYP76AK1* are highly correlated with *SmCYP76AH1*. These two *CYPs* could function sequentially to form a bifurcating pathway for tanshinone biosynthesis, converting ferruginol into 11,20-dihydroxy ferruginol and 11,20-dihydroxy sugiol^11^. The postmodification of ferruginol to tanshinones remains obscure, and CYPs, dehydrogenases and other enzymes are speculated to play important roles in this process.

CYPs are monooxygenases that are involved in numerous biosynthetic processes. Plant CYPs are the largest family of enzymes that contribute to the chemical diversity of secondary metabolites^10,12,13^. For example, the CYP71D subfamily is involved in the biosynthesis of terpenoids and flavonoids^12^. CYP72A1 is a secologanin synthase in the biosynthesis of terpene indole alkaloids in *Catharanthus roseus*^14^. The CYP76C subfamily is associated with monoterpenol metabolism in *Arabidopsis thaliana*^15^. In *S. miltiorrhiza*, CYPs in downstream tanshinone pathways have become a popular research field. SmCYP76AH1 is the first CYP that is responsible for the generation of oxygenated diterpenoid precursors in tanshinone biosynthesis^9^. It is always considered a marker gene for identifying possible genes involved in tanshinone biosynthesis postmodification. Gao *et al*. identified eight *CYPs* as targets for future investigation by transcriptomic analysis of *S. miltiorrhiza* hairy root culture^16^. Chen *et al*. identified 116 full-length and 135 partial-length *CYP*s in *S. miltiorrhiza*^17^. Coexpression and KEGG pathway analysis showed that three *CYP*s are potentially involved in terpenoid biosynthesis^17^. Xu *et al*. identified a total of 457 *CYPs* from the *S. miltiorrhiza* genome, and coexpression analysis suggested that 16 *CYPs* might be important in tanshinone biosynthesis^4^. Additionally, proteomic analysis also identified five CYPs as candidates that may be involved in tanshinone biosynthesis^18^. Other oxygenases and dehydrogenases may also be involved in tanshinone postmodification. The 2-oxoglutarate-dependent dioxygenase (2OGD) superfamily is a large gene family that is involved in the oxidic processes of natural products, such as terpenoid compounds. A total of 144 *2OGDs* have been identified in *S. miltiorrhiza*, and 16 *2OGDs* are more highly expressed in the periderm than in the rest of the root^4^. Recently, *Sm2OGD5* was found to play a crucial role in the downstream biosynthesis of tanshinones^19^. Moreover, dehydrogenases were suspected to play roles in tanshinone biosynthesis. A total of 159 short-chain alcohol dehydrogenases (SDRs) have been identified in *S. miltiorrhiza*, and five of them may play a role in tanshinone biosynthesis^4^. These studies provided candidate genes that may be involved in tanshinone biosynthesis for further investigation.

“99–3” and “shh” are two *S. miltiorrhiza* cultivars that are bred by Prof. Xianen Li at our institute. The genome of “99–3” has been sequenced and annotated^20^. In the present study, we performed transcriptome profiling analysis of two *S. miltiorrhiza* cultivars to investigate potential genes that are responsible for the third step of the tanshinone biosynthetic pathway. We analyzed immature roots, mature roots and mature root periderms in “99–3” and “shh” by transcriptome sequencing with three biological repeats. Differential expression analysis and weighted gene coexpression network analysis (WGCNA) were carried out to identify putative genes that are involved in the biosynthetic pathway of tanshinones. This study provides important insight for further investigation of tanshinone biosynthesis in *S. miltiorrhiza*.

## Results

### Transcriptome sequencing and assembly

**Figure 2:**
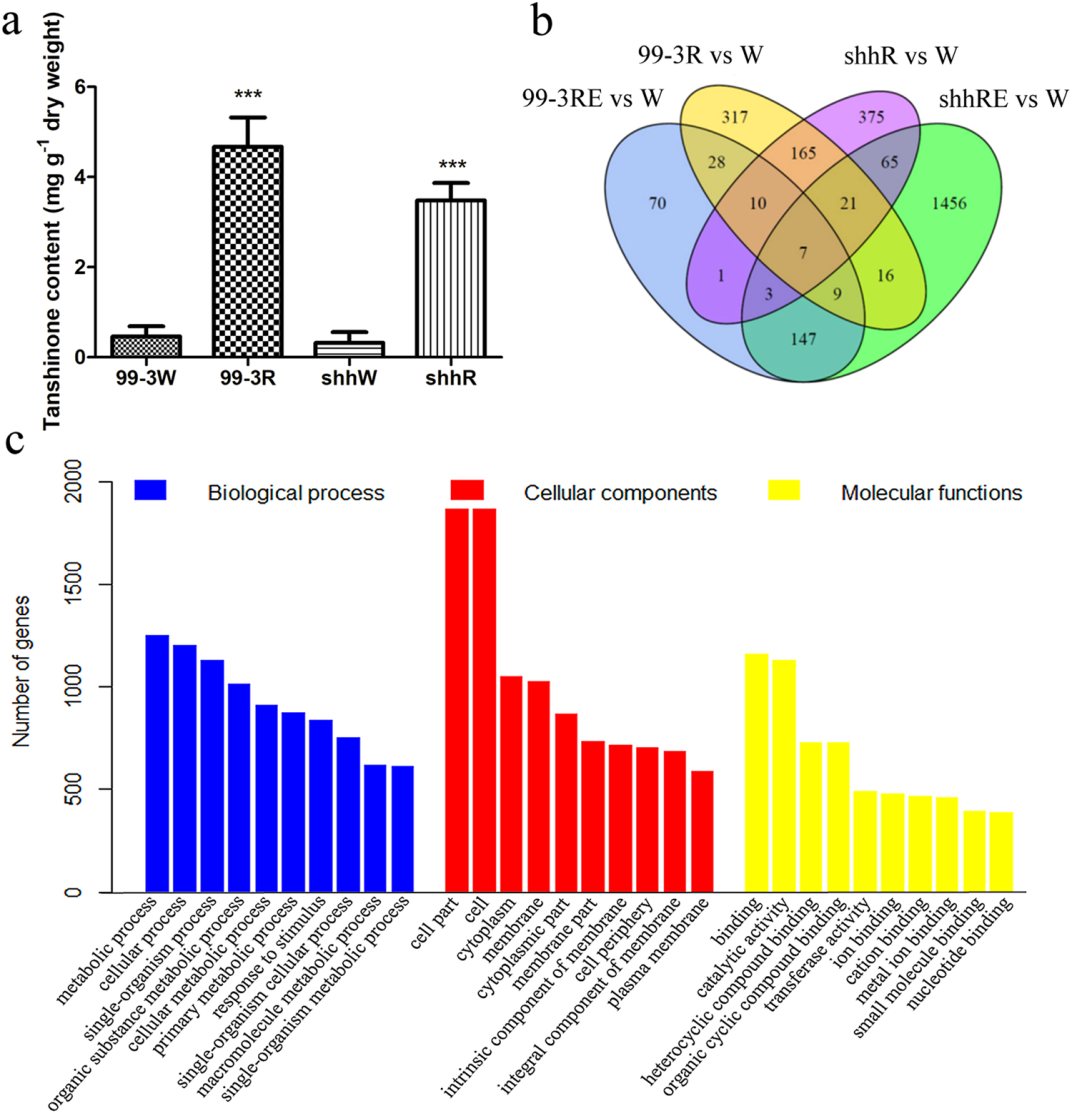
Tanshinone content of “99–3” and “shh” and expression analysis of differentially expressed genes (DEGs). (**a**) Tanshinone content in immature (99–3 W and shhW) and mature roots (99–3 R and shhR) of “99–3” and “shh”. Difference between mature roots and immature roots was analyzed by Student’s t-test. Significant difference (*p* < 0.001) was indicated by ***. (**b**) A venn diagram of DEGs in “99–3” and “shh”. (**c**) GO enrichment of DEGs.

Tanshinones mainly accumulate in the periderms of mature roots^4^. Analysis showed that the content of tanshinones in immature roots is significantly less than that in mature roots (Fig. 2a).

To investigate the downstream biosynthetic pathway of tanshinone, transcriptome sequencing was performed using the roots of “99–3” and “shh”, which are two cultivars of *S. miltiorrhiza*. Three biological replicates were analyzed for immature root (named 99–3 W or shhW), mature root (named 99–3 R or shhR) and mature root periderms (named 99–3RE or shhRE) of the two cultivars, and 18 samples were sequenced by the Illumina HiSeqTM 2500 sequencing platform. A total of 908.98 million raw reads were generated from these samples. After removing the adapter reads, reads with greater than 10% of unknown nucleotides and low-quality reads, 887.06 million clean pair-end reads were generated for assembly. Each sample yielded more than 5.30 Gb clean reads. The Q20 and Q30 percentages were more than 98% and 95%, respectively. The average GC content of the transcriptome was 47.93% (Supplementary Table S1). Mapping the clean reads from the 18 samples against the *S. miltiorrhiza* reference genome (“99–3”)^3^ showed that 621.69 million reads (70.43%) were mapped in total and 590.81 million reads (66.95%) were mapped uniquely. A *de novo* assembly of filtered reads was assembled into 35,466 unique genes. Among them, 30,478 genes (85.94%) were identified in a previous study^20^, whereas the other 4,988 genes were novel.

The expression values of all these genes were determined by reads per kilo bases per million reads (RPKM). To verify the reproducibility of the sequencing data, Person’s correlation coefficients for three biological replicates were calculated by log_10_(RPKM + 1). The results showed that all biological replicate correlations were greater than 0.85 (Supplementary Fig. S1). A total of 27,440 genes with an RPKM ≥ 1 in at least one of these samples were used for further analysis (Supplementary Table S2).

### Identification of differentially expressed genes

Since tanshinones mainly accumulate in the periderms of mature roots^4^, it is assumed that the expression levels of genes involved in tanshinone biosynthesis are significantly higher in mature roots than in immature roots. Two pairwise transcriptome comparisons were conducted to identify differentially expressed genes between mature roots (or periderms) and immature roots in both cultivars. Using a significance level of *p* < 0.05 and |log_2_(FoldChange)| > 1, we found 794 upregulated differentially expressed genes (DEGs) in 99–3 R (or 99–3RE) compared to 99–3 W and 2,275 upregulated DEGs in shhR (or shhRE) compared to shhW (Fig. 2b). Among them, 379 DEGs were redundant. Overall, a total of 2,690 DEGs with increased expression levels in mature roots compared with immature roots were identified. To gain more insight into the differential expression of these transcripts, hierarchical cluster and heat map analyses were performed based on the RPKM values. The results showed significant differential expression patterns (Supplementary Fig. S2). Except for 541 genes that were significantly downregulated in the periderms (99–3 RE and shhRE) than in roots (99–3 R and shhR), 2,149 DEGs were obtained. Among them, 1,104 genes were significantly up-regulated in the periderms (99–3RE and shhRE) compared with the roots (99–3R and shhR), suggesting they may have a periderm-specific profile. An additional 1,045 DEGs showed no significant difference between the periderms and roots, but they may contain genes with high expression levels in the periderms. Therefore, all 2,149 genes were further subjected to functional analysis.

To evaluate the major functional categories of DEGs, gene ontology (GO) enrichment was applied to identify the major functional categories represented by 2,149 DEGs. Among the biological process category, genes associated with metabolism, including metabolic process, organic substance metabolic process, cellular metabolic process, primary metabolic process, macromolecule metabolic process and single-organism metabolic process, were highly enriched (Fig. 2d). Additionally, DEGs were mapped to 118 KEGG pathways, and 20 significantly enriched pathways are shown in Table 1. The largest number of DEGs was annotated against biosynthesis of secondary metabolites and metabolic pathways. There are 22 genes associated with terpenoid backbone synthesis and 11 genes assigned to diterpenoid biosynthesis (Supplementary Table S3). The secondary metabolite biosynthesis pathway contains 17 functionally characterized tanshinone biosynthesis-related genes (Supplementary Table S3). These results suggest that the DEGs enriched in secondary metabolite biosynthesis may provide new information for analysis of tanshinone biosynthesis in *S.miltiorrhiza*.

**Table 1:** KEGG pathway enrichment analysis of genes that are differentially expressed between mature roots and immature roots in two cultivars.

Term	ID	*p*-value	Gene number
**Biosynthesis of secondary metabolites**	ath01110	2.01E-22	203
Metabolic pathways	ath01100	3.69E-17	282
Phenylpropanoid biosynthesis	ath00940	5.53E-12	48
Stilbenoid, diarylheptanoid and gingerol biosynthesis	ath00945	1.28E-09	23
Flavonoid biosynthesis	ath00941	5.45E-09	16
Starch and sucrose metabolism	ath00500	1.04E-07	45
**Terpenoid backbone biosynthesis**	ath00900	1.47E-07	22
Glutathione metabolism	ath00480	5.34E-07	27
Amino sugar and nucleotide sugar metabolism	ath00520	8.79E-07	33
Sesquiterpenoid and triterpenoid biosynthesis	ath00909	1.79E-06	13
Limonene and pinene degradation	ath00903	1.11E-05	16
Pentose and glucuronate interconversions	ath00040	3.92E-05	21
Cyanoamino acid metabolism	ath00460	0.001733	14
Ether lipid metabolism	ath00565	0.005031	8
Phenylalanine metabolism	ath00360	0.006708	10
Phenylalanine, tyrosine and tryptophan biosynthesis	ath00400	0.007249	12
Monoterpenoid biosynthesis	ath00902	0.008082	4
Pentose phosphate pathway	ath00030	0.018543	11
**Diterpenoid biosynthesis**	ath00904	0.019843	11
Carbon fixation in photosynthetic organisms	ath00710	0.024463	12

### Weighted gene coexpression network analysis

To further investigate genes that are related to the biosynthesis of tanshinones, highly coexpressed gene modules were inferred from all genes of 18 samples (three biological replicates) using WGCNA^21^. Highly interconnected genes clustered in the same modules were labeled by different colors. In each module, genes were coexpressed and functionally related. Therefore, we identified a total of 38 coexpression modules, and the 2,149 DEGs were clustered into 36 modules. (Supplementary Fig. S3). The majority of these DEGs were grouped into the “purple” and “cyan” modules that contained 24.71% (531) and 18.66% (401) of the total DEGs, respectively. The other 34 modules comprised 0.05–9.77% of the total DEGs (Table 2).

**Table 2:** Weighted gene coexpression network analysis (WGCNA) of DEGs.

Module name	DEGs		Tanshinone biosynthesis related genes and their homologous genes		
	Number of genes	% of total	Number of genes	% of total	Genes
cyan	401	18.66%	19	33.33%	*SmCYP76AH1* *, SmCYP76AH3v1 *, SmCYP76AH3v2 *, SmCYP76AK1 *, SmGGPPS1 *, SmKSL1 *, SmCPS1 *, SmCPR1 *, SmCPR2 *, SmDXR *, SmHDR1 *, SmCMK *, SmMCT *, SmMDS *, *SmHMGR1* *, SmDXS2, SmGPPS.SSUII.1 SmHDR3, SmHMGR4
midnightblue	210	9.77%	6	10.53%	*SmGGPPS3, SmHMGS1, SmHMGS2, SmIDI1* *, SmMDC *, SmPMK *
blue	54	2.51%	6	10.53%	*SmAACT6, SmDXS1* *, SmGPPS.SSUI1, SmGPPS.SSUII.3, SmHDR2, SmHDR4
greenyellow	109	5.07%	4	7.02%	*SmGPPS.SSUI2, SmIDI3, SmIDI4, SmIDI5*
turquoise	58	2.70%	4	7.02%	*SmAACT2,SmAACT3,SmAACT5,SmCPS9*
purple	531	24.71%	3	5.26%	*SmAACT1* *, SmCPS5, SmHMGR2
brown	95	4.42%	2	3.51%	*SmCPS6, SmGPPS.SSUII.2*
yellow	20	0.93%	2	3.51%	*SmDXS5, SmKSL2*
tan	2	0.09%	2	3.51%	*SmCPS3, SmCPS8*
darkturquoise	21	0.98%	1	1.75%	*SmDXS4*
red	62	2.89%	1	1.75%	*SmAACT4*
darkgrey	31	1.44%	1	1.75%	*SmCPS4*
darkred	13	0.60%	1	1.75%	*SmDXS3*
yellowgreen	22	1.02%	1	1.75%	*SmMK* *
salmon	7	0.33%	1	1.75%	*SmGGPPS2*
paleturquoise	7	0.33%	1	1.75%	*SmCPS2*
violet	3	0.14%	1	1.75%	*SmKSL3*
green	110	5.12%	\	\	*\*
black	157	7.31%	\	\	\
grey60	41	1.91%	\	\	\
darkmagenta	22	1.02%	\	\	\
lightcyan	49	2.28%	\	\	\
darkolivegreen	32	1.49%	\	\	\
lightgreen	16	0.74%	\	\	\
lightyellow	17	0.79%	\	\	\
darkorange	17	0.79%	\	\	\
magenta	9	0.42%	\	\	\
sienna3	7	0.33%	\	\	\
white	7	0.33%	\	\	\
steelblue	5	0.23%	\	\	\
pink	5	0.23%	\	\	\
saddlebrown	4	0.19%	\	\	\
darkgreen	1	0.05%	\	\	\
orange	2	0.09%	\	\	\
royalblue	1	0.05%	\	\	\
skyblue	1	0.05%	\	\	\
TOTAL	2149		57		

*Tanshinone biosynthesis-related genes reported in previous studies^2,4–11^.

Further analysis showed that functionally characterized tanshinone biosynthesis related genes and their homologous genes^4,6^ were distributed in 18 modules (Table 2). The “cyan” module contains 15 previously reported genes that are involved in the tanshinone biosynthesis pathway (Table 2). This result indicated that genes in the “cyan” module were most likely associated with tanshinone biosynthesis. Further analysis was performed for the “cyan” module genes.

### Potential genes related to tanshinone biosynthesis

**Figure 3:**
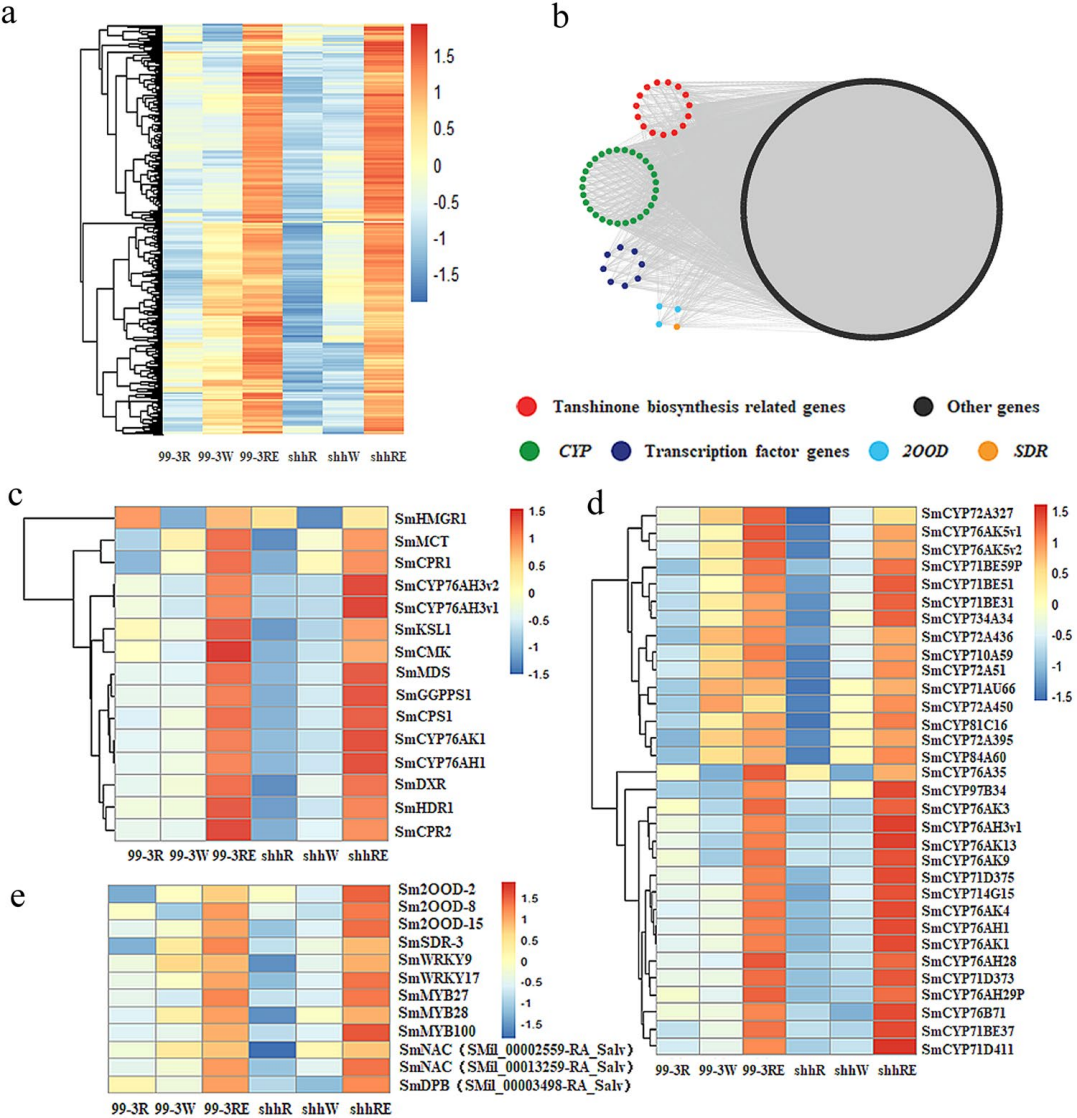
Coexpression network analysis in the “cyan” module. (**a**) A heatmap of coexpressed genes in the “cyan” module. (**b**) Cytoscape representation of coexpressed genes with edge weights > 0.1 in the “cyan” module. The red, green, darkblue, lightblue, yellow and black nodes represent tanshinone biosynthesis-related genes, *CYP* genes, transcription factor genes, *2OGD*, *SDR* and other genes, respectively. (**c**) Expression patterns of the tanshinone biosynthesis-related genes. (**d**) Expression patterns of *CYPs* in the “cyan” module. (**e**) Expression patterns of *2OGDs*, *SDR* and transcription factor genes in the “cyan” module.

As mentioned above, the “cyan” module most likely contains tanshinone biosynthesis-related candidate genes. The expression patterns showed that the majority of genes in this module had similar expression patterns and were specifically expressed in the mature root periderms (Fig. 3a). We visualized the coexpression networks of this module using Cytoscape^22,23^, and each node represents a gene and the connecting lines (edges) between genes represent coexpression correlations (Fig. 3b). Hub genes are genes with the most connections in a network. Thirty-three hub genes were selected from the “cyan” module (Supplementary Table S4). They included two key genes (*SmMDS* and *SmGGPPS1*) in the tanshinone biosynthesis pathway, further verifying our gene identification method. This result suggests that the other 31 hub genes might be associated with tanshinone biosynthesis.

Since *CYPs* have been proven to play an important role in secondary metabolic biosynthesis, we focused on the 29 *CYP* genes in the “cyan” module (Table 3). They were classified into nine families and 14 subfamilies, and the CYP76, CYP71 and CYP72 families contained ten, eight and five members, respectively. The other members belonged to the CYP710, CYP714, CYP734, CYP81, CYP84 and CYP97 families. All these *CYPs* have high expression levels in the periderms, similar to the expression levels of key enzyme genes in tanshinone biosynthesis (Fig. 3d). To better understand the putative functions of CYPs, we constructed a phylogenetic tree with their amino acid sequences. The tree showed that all the 29 CYPs were distributed into three clades. The CYP76 family members were clustered into one clade. SmCYP84A60 and the CYP71 family members were grouped into another clade. The third clade contained CYP72 family members together with SmCYP81C16, SmCYP97B34, SmCYP710A59, SmCYP714G15 and SmCYP734A34 (Supplementary Fig. S4). The CYP76 family, which contains three key enzyme genes involved in the third stage of tanshinone biosynthesis^9,11^, was further analyzed. Both *SmCYP76AH28* and *SmCYP76AH29P* belong to the CYP76AH subfamily. However, sequence analysis found that *SmCYP76AH28* is a partial sequence of *SmCYP76AH3* with only 4 bp nucleotide mutations (Supplementary Fig. S5 and Table S5). SmCYP76AH29P shares a 72.80% amino acid sequence identity with SmCYP76AH1 and an 88.80% identity with SmCYP76AH3, suggesting that it may have functions similar to SmCYP76AH1 and SmCYP76AH3 (Supplementary Fig. S5 and Table S5). SmCYP76AK3, SmCYP76AK4, SmCYP76AK5v1, SmCYP76AK5v2, SmCYP76AK9 and SmCYP76AK13 are members of the CYP76AK subfamily, which share 69.78%, 63.62%, 69.18%, 66.02%, 37.38% and 69.18% amino acid sequence identity with SmCYP76AK1, respectively (Supplementary Fig. S6 and Table S5). These CYP76AKs may also participate in tanshinone biosynthesis. Moreover, the CYP71 and CYP72 families were shown to be associated with terpene metabolite biosynthesis. It has been reported that the CYP71D subfamily is involved in the biosynthesis of terpenoids and flavonoids in *Arabidopsis*^12^. CYP72A1 is a secologanin synthase in the biosynthesis of terpene indole alkaloids in *Catharanthus roseus*^14^. Therefore, the eight *CYP71s* and five *CYP72s* may also warrant further investigation. GO and KEGG pathway analysis showed that 11 *CYPs*, including six members of the CYP71 family (*SmCYP71BE31*, *SmCYP71BE51*, *SmCYP71BE59P*, *SmCYP71D373*, *SmCYP71D375* and *SmCYP71D411*), three members of the CYP76 family (*SmCYP76A35*, *SmCYP76AK3* and *SmCYP76B71*), one member of the CYP710 family (*SmCYP710A59*) and one member of the CYP714 family (*SmCYP714G15*), are annotated against biosynthesis of secondary metabolites (Table 3 and Supplementary Table S3). Among them, *SmCYP76A35* and *SmCYP714G15* are also hub genes in the coexpression module (Supplementary Table S4), suggesting that they may play a central role in the module. GO and KEGG analysis also indicated that *SmCYP734A34*, *SmCYP84A60* and *SmCYP97B34* are associated with brassinosteroid biosynthesis, phenylpropanoid biosynthesis and carotenoid biosynthesis, respectively (Table 3). They were not considered to be involved in tanshinone biosynthesis. Overall, we identified 25 *CYPs*, of which 14 *CYPs* were previously reported^4^ and 11 *CYPs* were identified in this study, that may be involved in tanshinone biosynthesis.

**Table 3:** A list of *CYPs* in *S. miltiorrhiza* identified in the “cyan” module.

Gene ID	Gene name	GO & KEGG pathway	CYP family	CYP subfamily
SMil_00022912-RA_Salv	***SmCYP710A59*** ^#^	biosynthesis of secondary metabolites	CYP710	CYP710A
SMil_00010521-RA_Salv	***SmCYP714G15*** ^#^ *	biosynthesis of secondary metabolites	CYP714	CYP714G
SMil_00019862-RA_Salv	SmCYP71AU66 *	biosynthesis of secondary metabolites	CYP71	CYP71AU
SMil_00028004-RA_Salv	***SmCYP71BE31*** ^#^ *	biosynthesis of secondary metabolites		CYP71BE
SMil_00021803-RA_Salv	SmCYP71BE37 *	\		
SMil_00005904-RA_Salv	***SmCYP71BE51*** ^#^ *	biosynthesis of secondary metabolites		
SMil_00026083-RA_Salv	***SmCYP71BE59P*** ^#^	biosynthesis of secondary metabolites		
SMil_00024176-RA_Salv	***SmCYP71D373*** ^#^ *	biosynthesis of secondary metabolites		CYP71D
SMil_00024363-RA_Salv	***SmCYP71D375*** ^#^ *	biosynthesis of secondary metabolites		
SMil_00004793-RA_Salv	***SmCYP71D411*** ^#^ *	biosynthesis of secondary metabolites		
SMil_00014210-RA_Salv	***SmCYP72A436***	\	CYP72	CYP72A
SMil_00014211-RA_Salv	***SmCYP72A327***	metabolic process		
SMil_00018783-RA_Salv	SmCYP72A395 *	metabolic process		
SMil_00001696-RA_Salv	***SmCYP72A450***	metabolic process		
SMil_00011721-RA_Salv	***SmCYP72A451***	\		
SMil_00021095-RA_Salv	*SmCYP734A34*	brassinosteroid biosynthesis	CYP734	CYP734A
SMil_00024737-RA_Salv	***SmCYP76A35*** ^#^	biosynthesis of secondary metabolites	CYP76	CYP76A
SMil_00020971-RA_Salv	*SmCYP76AH28*	\		CYP76AH
SMil_00006344-RA_Salv	***SmCYP76AH29P***	\		
SMil_00006784-RA_Salv	***SmCYP76AK13***	\		CYP76AK
SMil_00017381-RA_Salv	***SmCYP76AK3*** ^#^ *	biosynthesis of secondary metabolites		
SMil_00026561-RA_Salv	SmCYP76AK4 *	metabolic process		
SMil_00029369-RA_Salv	SmCYP76AK5v1 *	\		
SMil_00029649-RA_Salv	SmCYP76AK5v2 *	\		
SMil_00017382-RA_Salv	***SmCYP76AK9***	\		
SMil_00026342-RA_Salv	***SmCYP76B71***	\		CYP76B
SMil_00022797-RA_Salv	SmCYP81C16 *	\	CYP81	CYP81C
SMil_00026853-RA_Salv	*SmCYP84A60*	phenylpropanoid biosynthesis	CYP84	CYP84A
SMil_00021903-RA_Salv	*SmCYP97B34*	carotenoid biosynthesis	CYP97	CYP97B

*CYPs* identified as candidate genes in the tanshinone biosynthesis pathway are highlighted in bold. ^**#**^ genes mapped in biosynthesis of secondary metabolites. * reported in a previous study^4^.

Considering the highly oxidized nature of tanshinones, other oxygenases and dehydrogenases may also be involved in tanshinone biosynthesis. 2OGDs and SDRs were suspected to play roles in tanshinone production^4^. In the “cyan” module, we identified three *2OGDs* (*Sm2OGD-2*, *Sm2OGD-8* and *Sm2OGD-15*) and one dehydrogenase gene (*SmSDR-3*) (Fig. 3e). They were highly expressed in the periderm, which is consistent with previous reports^4^. In particular, *2OGD-8* and *SDR-3* have been reported to exhibit a periderm-specific expression profile^4^. KEGG analysis indicated that *2OGD-15* was associated with diterpenoid biosynthesis (Supplementary Table S3). Thus, these genes were also considered to be candidates that may be related to tanshinone biosynthesis.

Some studies have shown that transcription factors could promote tanshinone accumulation^24,25^, but the regulatory mechanism remains unelucidated. In this study, eight transcription factors were identified and classified into four transcription factor families, including two WRKY transcription factors (SmWRKY9 and SmWRKY17)^26^, three MYB transcription factors (SmMYB27, SmMYB28 and SmMYB100)^27^, two putative NAC transcription factors and one putative DPB transcription factor. They were specifically expressed in the periderm (Fig. 3e), suggesting their putative roles in tanshinone biosynthesis.

### Expression analysis of selected candidate genes in response to MeJA treatment

For the postmodification of tanshinone, CYPs, dehydrogenases and other enzymes are speculated to play important roles. As mentioned above, we identified 25 *CYPs*, three *2OGDs* and one *SDR* that may be involved in this process. Among them, 14 *CYPs*, three *2OGDs* and one *SDR* have reported in a previous study^4^. Therefore, the remaining 11 *CYPs*, including *SmCYP710A59*, *SmCYP71BE59P*, *SmCYP72A436*, *SmCYP72A327*, *SmCYP72A450*, *SmCYP72A451*, *SmCYP76A35*, *SmCYP76AH29P*, *SmCYP76AK9*, *SmCYP76AK13* and *SmCYP76B71*, were further analyzed experimentally.

**Figure 4:**
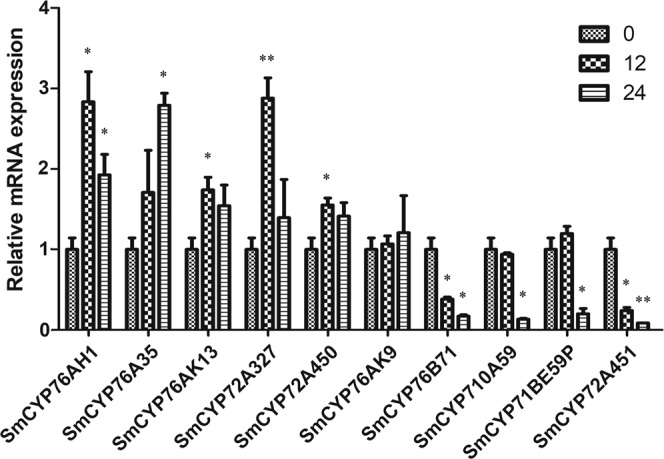
Fold changes of CYPs in the roots of *S. miltiorrhiza* after MeJA treatment for 12 and 24 h. The expression levels were analyzed by qRT-PCR. CYP expression at 0 h were considered the controls. The values are representative of three biological replicates. Significant differences between MeJA treatment for 12 or 24 h and the control (0 h) were determined by Student’s t-test (*p < 0.05 and **p < 0.01).

Methyl jasmonate (MeJA) is an effective elicitor that enhances the accumulation of tanshinone in *S. miltiorrhiza*^28–30^. We analyzed the expression of 11 *CYP* candidates in *S. miltiorrhiza* roots treated with 200 μM exogenous MeJA for 0, 12 and 24 h using qRT-PCR. The results showed that the expression levels of *SmCYP76A35*, *SmCYP76AK13*, *SmCYP72A327* and *SmCYP72A450* were all upregulated in roots treated with MeJA for 12 and 24 h (Fig. 4). Their expression profiles in response to MeJA were similar to those of *SmCYP76AH1*. No significant changes were observed in the expression level of *SmCYP76AK9* after MeJA treatment. Additionally, the expression levels of *SmCYP76B71*, *SmCYP710A59*, *SmCYP71BE59P* and *SmCYP72A451* were all decreased after MeJA treatment (Fig. 4). The expression of *SmCYP72A436* and *SmCYP76AH29P* was not detected in the roots. All these results indicated that *SmCYP76A35*, *SmCYP76AK13*, *SmCYP72A327* and *SmCYP72A450* were upregulated by MeJA. These CYP450s can be selected for further investigation for their involvement in tanshinone biosynthesis.

## Discussion

Tanshinones, which are the main bioactive components of *S. miltiorrhiza*, mainly accumulate in the periderms of mature roots^4^. Elucidating the biosynthesis of tanshinones and identifying the key enzyme genes in the biosynthetic pathway are necessary for improving the production of tanshinones.

WGCNA, which is one of the most reliable and effective methods for the analysis of gene functions^21^, using a large sample size ( ≥ 15) could comprehensively identify relationships between individual genes. This method has been successfully used in strawberry^31^, *Prunus salicina*^32^, rice^33^, lotus^34^, *etc*. In this study, we identified a “cyan” module that may be related to tanshinone biosynthesis by differential expression analysis and WGCNA. This module contained all the reported functionally characterized genes in the third stage of tanshinone biosynthesis. Among the 25 *CYPs* identified in the “cyan” module, 14 *CYPs* were consistent with those reported in a previous study^4^. All these results confirmed that our candidate genes were reliable.

The third stage of tanshinone biosynthesis is complex, and the postmodification from ferruginol to tanshinones remains obscure. CYPs, dehydrogenases, demethylases, and other enzymes are speculated to play roles in this process^2^. In this study, we also found that 25 *CYPs*, including 11 newly identified *CYPs*, might be involved in tanshinone biosynthesis by differential expression analysis and WGCNA (Fig. 3). The majority of the 25 candidate *CYPs* belong to the CYP76, CYP71 and CYP72 families (Table 3).

For the 14 previously reported CYPs^4^, our results also indicated that they were specifically expressed in the periderms, which was consistent with a previous study (Fig. 3). Eight *CYPs*, including *SmCYP76AK3*, *SmCYP76AK4*, *SmCYP76AK5v1*, *SmCYP76AK5v2*, *SmCYP71D373*, *SmCYP71D375*, *SmCYP71D411* and *SmCYP71AU66*, were reported to be specifically expressed in the periderm^4^. Among these *CYPs*, GO and KEGG analysis showed that *SmCYP71D373*, *SmCYP71D375*, *SmCYP71D411* and *SmCYP76AK3* are associated with the biosynthesis of secondary metabolites (Table 3), implying that they are most likely to be involved in tanshinone biosynthesis. Proteomics analysis revealed five new *CYPs* as candidates that are involved in tanshinone biosynthesis^18^. In this study, sequencing analysis indicated that one of these *CYPs* is *SmCYP76AK5v1* or *SmCYP76AK5v2*. Thus, *SmCYP76AK5v1* and *SmCYP76AK5v2* warrant further investigation. Moreover, *SmCYP714G15* was identified as the hub gene in the tanshinone biosynthesis-related module and was annotated against the biosynthesis of secondary metabolites (Table 3, Supplementary Table S3 and S4), indicating that it may also be considered a candidate gene in tanshinone biosynthesis.

For the other 11 identified *CYPs*, *SmCYP76A35*, *SmCYP76AK13*, *SmCYP72A327* and *SmCYP72A450* were considered candidates that may be involved in tanshinone biosynthesis. *SmCYP76A35* was the hub gene in the “cyan” module, which was associated with the biosynthesis of secondary metabolites (Supplementary Table S3, S4). The expression of *SmCYP76A35* was enhanced by MeJA (Fig. 4), revealing its possible roles in the tanshinone biosynthesis pathway. The expression level of *SmCYP76AK13* was also significantly increased after treatment with MeJA (Fig. 4). SmCYP76AK13, which shares 69.18% amino acid sequence identity with SmCYP76AK1, belongs to the CYP76AK subfamily (Supplementary Fig. S6 and Table S5), suggesting that it may have a similar function to SmCYP76AK1. Some CYP72A subfamily members were reported to be associated with the biosynthesis of secondary metabolites. CYP72A1 from *Catharanthus roseus* is a secologanin synthase in the biosynthesis of the seco-iridoid unit of terpene indole alkaloids^14^. CYP72A67 of *Medicago truncatula*^35^ and CYP72A154 of *Glycyrrhiza uralensis*^36^ have been reported to participate in oleanane triterpene scaffold oxidation. We also found that two CYP72A subfamily members, which include *SmCYP72A327* and *SmCYP72A450*, were coexpressed with tanshinone biosynthesis-related genes and could be upregulated upon application of MeJA (Fig. 4), suggesting a possible role in tanshinone biosynthesis.

Two CYP76AH subfamily genes, which include *SmCYP76AH28* and *SmCYP76AH29P*, were analyzed in this study. Sequence analysis showed that *SmCYP76AH28* is a partial sequence of *SmCYP76AH3* and that SmCYP76AH29P shares 88.80% identity with SmCYP76AH3 (Supplementary Fig. S5 and Table S5). Due to its highly similar sequence to *SmCYP76AH3*, the expression level of *SmCYP76AH29P* was analyzed using a specific region. However, the expression of *SmCYP76AH29P* in the roots was not detected, likely because the specific region of *SmCYP76AH29P* may not exist. This result suggested that *SmCYP76AH28* and *SmCYP76AH29P* may be generated by incorrect sequence assembly and may be the same gene as *SmCYP76AH3*.

2OGDs and SDRs were suspected to play roles in tanshinone production^4^. A total of 144 *2OGDs* have been identified in *S. miltiorrhiza*, and 16 of these genes are more highly expressed in the periderm^4^. *2OGD5* was found to play a crucial role in the downstream biosynthesis of tanshinones^19^. Moreover, 159 *SDRs* were identified in *S. miltiorrhiza*, and five of them may be associated with tanshinone biosynthesis^4^. In this study, we found that three *2OGDs* and one *SDR* may be involved in tanshinone biosynthesis. They were all highly expressed in the periderm (Fig. 3) and consistent with a previous study^4^. In particular, *Sm2OGD-8* and *SmSDR-3* were reported to exhibit a periderm-specific expression profile^4^. *Sm2OGD-15* was assigned to diterpenoid biosynthesis by KEGG analysis (Supplementary Table S3). Therefore, *Sm2OGD-8*, *Sm2OGD-15* and *SmSDR-3* should be prioritized in further investigations of tanshinone biosynthesis.

Various transcription factors are involved in tanshinone biosynthesis by *S. miltiorrhiza*, but studies on transcription factors that regulate tanshinone biosynthesis are limited. The *MYB* gene family is the largest transcription factor family, and 110 *SmMYB* genes have been characterized in *S. miltiorrhiza*^27^. The overexpression of *SmMYB36* in the hairy roots could promote tanshinone accumulation^37^. *SmMYB9b* could enhanced tanshinone concentration by stimulating the MEP pathway^24^. It was reported that 61 *SmWRKYs* were cloned and characterized from *S. miltiorrhiza*, and systematic analysis suggested that *SmWRKY3* and *SmWRKY9* are most likely activators in tanshinone biosynthesis^25^. Overexpression of *SmWRKY2* in *S. miltiorrhiza* hairy roots significantly increases the accumulation of tanshinones^38^. A total of 127 *bHLH* transcription factor genes have been identified in *S. miltiorrhiza*, and 7 of them are potentially associated with the regulation of tanshinone biosynthesis^25^. *SmbHLH10* could improve tanshinone production in *S. miltiorrhiza* hairy roots by activating the expression of the key enzyme genes involved in tanshinone biosynthesis^39^. Two AP2/ERF transcription factors, SmERF1L1 and SmERF128, have been reported to positively regulate tanshinone biosynthesis in *S. miltiorrhiza*^40^. Through DEG and WGCNA analysis, we also found that eight transcription factor genes might be involved in tanshinone biosynthesis (Fig. 3). They have a similar expression pattern and are coexpressed with tanshinone biosynthesis-related genes (Fig. 3). Among them, *SmWRKY9*, *SmWRKY17*, *SmMYB27* and *SmMYB100* were shown to be most highly expressed in roots^26,27^. The function of these transcription factors in tanshinone biosynthesis requires further confirmation.

In conclusion, we identified a “cyan” module that is associated with tanshinone biosynthesis by differential expression analysis and WGCNA. In this module, 25 *CYPs*, three *2OGDs*, one *SDR* and eight transcription factors that they may be involved in tanshinone biosynthesis were identified. For the 25 *CYPs*, 14 *CYPs* have been reported previously^4^, whereas the other 11 *CYPs* were identified in this study. Expression analysis showed that four newly identified *CYPs* are upregulated upon the application of MeJA, suggesting their possible roles in tanshinone biosynthesis. Overall, the candidate genes involved in tanshinone biosynthesis that were identified in this study provide insight for future research. The function of these genes in the tanshinone biosynthesis pathway should be further investigated.

## Materials and Methods

### Plant materials

Two cultivars of *S. miltiorrhiza*, which included “99–3” and “shh”, were harvested from an experimental field in late August at the Institute of Medicinal Plant Development (IMPLAD). Root samples from “99–3” and “shh” were collected and named 99–3/shhW (immature roots), 99–3/shhR (mature roots) and 99–3/shhRE (mature root periderms). Immature roots are smaller roots without a tanshinone color, while mature roots are well-developed roots with a tanshinone color (Supplementary Fig. S7). Biological replicates were obtained from three independent plants.

### Analysis of tanshinone content by HPLC-UV

Tanshinone content was analyzed as described in the Pharmacopoeia of the People’s Republic of China (2015)^41^. Three biological repeats were performed. Dried mature roots of “99–3” and “shh” were ground into a powder, and then each sample of weighed ground powder (0.3 g) was extracted with 50 ml of methanol. After 30 min of ultrasonic extraction, methanol was added to complement and maintain a constant weight. The sample was filtrated with 0.22 μm filter membrane, and then analyzed immediately using HPLC-UV. To determine tanshinone content, 10 μL of extraction product was injected into a Supelco C18 HPLC column (4.6 × 250 mm) and analyzed by HPLC-UV under the following conditions. Solvent system: methanol/H_2_O (75:25); flow rate: 0.8 mL/min; temperature: 25 °C; detection: absorbance measured at 270 nm. Tanshinone I, tanshinone IIA, cryptotanshinone and dihydrotanshinone standards were dissolved in methanol at a concentration of 0.04, 0.03, 0.02, and 0.02 mg.mL^−1^. Total content of tanshinone I, tanshinone IIA, cryptotanshinone and dihydrotanshinone in each sample was calculated.

### RNA sequencing

For transcriptome sequencing, a total of 18 RNA samples were extracted by the Quick RNA isolation kit (Huayue Yang, Beijing, China) according to the manufacturer’s instructions. RNA degradation and contamination was monitored on 1% agarose gels. RNA purity was checked using the NanoPhotometer spectrophotometer (IMPLEN, CA, USA). RNA concentration was measured using the Qubit RNA Assay Kit Qubit 2.0 Flurometer (Life Technologies, CA, USA). RNA integrity was assessed using the RNA Nano 6000 Assay Kit and the Bioanalyzer 2100 system (Agilent Technologies, CA, USA). Sequencing libraries were generated using the NEBNext Ultra™ RNA Library Prep Kit for Illumina® (NEB, USA). The library preparations were sequenced on an Illumina Hiseq. 2500 platform, and 150 bp paired-end reads were generated.

### RNA-seq data analysis

Clean data (clean reads) were obtained by removing reads containing adapters, reads containing ploy-N and low quality reads from raw data. Q20, Q30 and GC content of the clean data were calculated. All downstream analyses were based on high-quality clean data.

A reference genome and gene model annotation files were downloaded from http://www.ndctcm.org/shujukujieshao/2015-04-23/27.html^20^. The index of the reference genome was built using Bowtie v2.0.6 software, and paired-end clean reads were aligned to the reference genome using TopHat v2.0.9. HTSeq v0.6.1 was used to count the read numbers that were mapped to each gene. The RPKM of each gene was calculated based on the length of the gene and the read count mapped to this gene. The Cufflinks v2.1.1 reference annotation-based transcript (RABT) assembly method was used to construct and identify known and novel transcripts from the TopHat alignment results.

### Differential expression analysis

Differential expression analysis was performed using the DESeq R package (1.10.1). The resulting *p*-values were adjusted using Benjamini and Hochberg’s approach for controlling the false discovery rate. Genes identified by DESeq with an adjusted *p*-value < 0.05 were considered differentially expressed. Venn diagram is conducted by the Venndiagram R package.

### GO and KEGG enrichment analysis

Gene ontology (GO) enrichment analysis of differentially expressed genes was conducted by the GOseq R package, and the gene length bias was corrected. GO terms with a corrected *p*-value < 0.05 were considered significantly enriched by differential expression analysis. To identify the functions of DEGs, KEGG pathway enrichment analysis of the DEGs was performed using KOBAS software (http:// kobas.cbi.pku.edu.cn).

### Gene coexpression network analysis

WGCNA can be used to identify modules of highly correlated genes and hub genes with important effects^21^. Gene expression was also evaluated with the WGCNA R package version 1.34^21^. We first combined the expression matrix of 27,440 genes as the input file for WGCNA analysis to identify modules of genes with strong co-expression. Next, WGCNA network construction and module detection were conducted using an unsigned type of topological overlap matrix (TOM). The soft power value is 18, minModuleSize is 30, and mergeCutHeight is 0.25. The most significantly correlated genes with a WGCNA edge weight > 0.1 were visualized using Cytoscape 3.5.1 software^22,23^. The phylogenetic tree was constructed using the Neighbor-Joining (NJ) method in MEGA6.

### MeJA treatment and qRT-PCR analysis

Healthy *S. miltiorrhiza* plantlets (99–3 line) were subcultivated on Murashige and Skoog (MS) agar medium for 5 weeks under a 16/8 h light/dark photoperiod at 25 °C and then transferred to MS liquid medium for 2 days. Then, MeJA was added to the medium at a final concentration of 200 μM. Roots were collected at 0, 12 and 24 h after treatment. Three biological replicates were performed for each treatment.

Total RNA was extracted from the roots using an RNAprep Pure Plant Kit (Axygen, Beijing, China). Then, first-strand cDNA was synthesized with AMV RT (Invitrogen, CA, USA). qRT-PCR was performed using 100 ng cDNA, 10 μL of SYBR Premix Ex Taq (TaKaRa, Otsu, Japan) and 0.5 μM of each primer in a 20 μL volume. The transcription products were amplified using different primers (Supplementary Table S6). A standard 2-step protocol was followed: enzyme activation 10 min at 95 °C, followed by 40 cycles of 95 °C for 15 s, 60 °C for 45 s. The *S. miltiorrhiza UBQ* gene was used as a control for qRT-PCR reactions. The PCR experiment was repeated three times.

## Supplementary information

supplementary information
