# Supplementary material for: Transcriptomic analysis reveals potential genes involved in tanshinone biosynthesis in Salvia miltiorrhiza
Source: Sci Rep. 2019 Oct 17;9:14929. doi: 10.1038/s41598-019-51535-9 (PMC6797793; doi:10.1038/s41598-019-51535-9)
Supplement: Supplementary file 1 — supplementary information [file 41598_2019_51535_MOESM1_ESM.docx]

**Transcriptomic analysis reveals potential genes involved in tanshinone biosynthesis in *Salvia miltiorrhiza***

Yujie Chang^1,2^, Meizhen Wang^1^, Jiang Li^3^, Shanfa Lu^1 *^

**Supplementary Information**


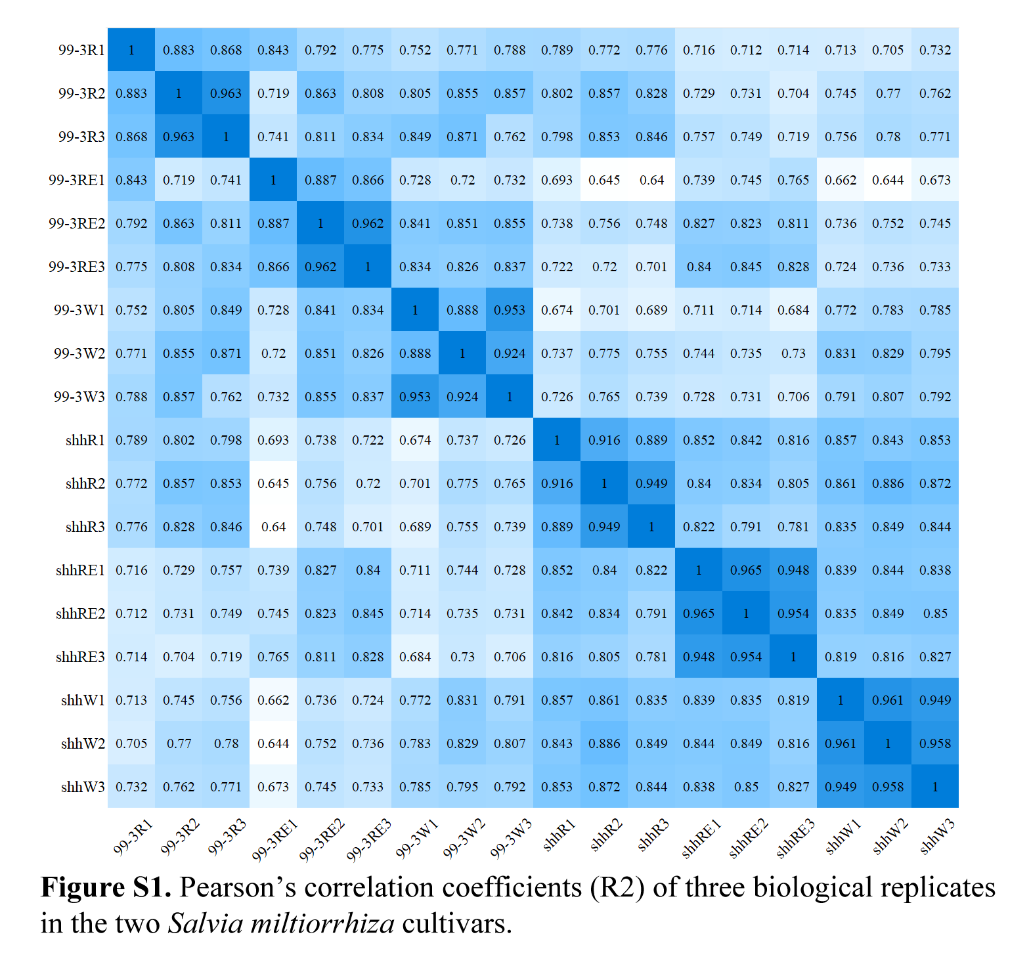


**Supplementary Figure S1.** Pearson’s correlation coefficients (R^2^) of three biological replicates in the two cultivars.


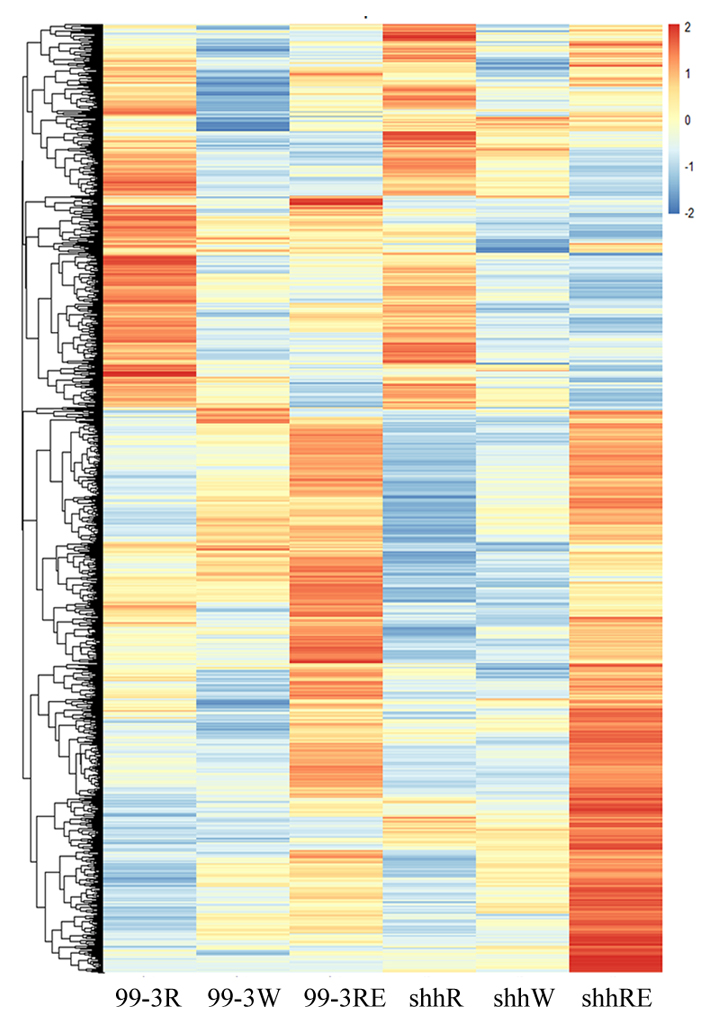


**Supplementary Figure S2.** A heatmap of the expression of all DEGs in different samples. The heatmap colors represent gene expression values calculated by log_2_(RPKM+1) based on the provided color scale.


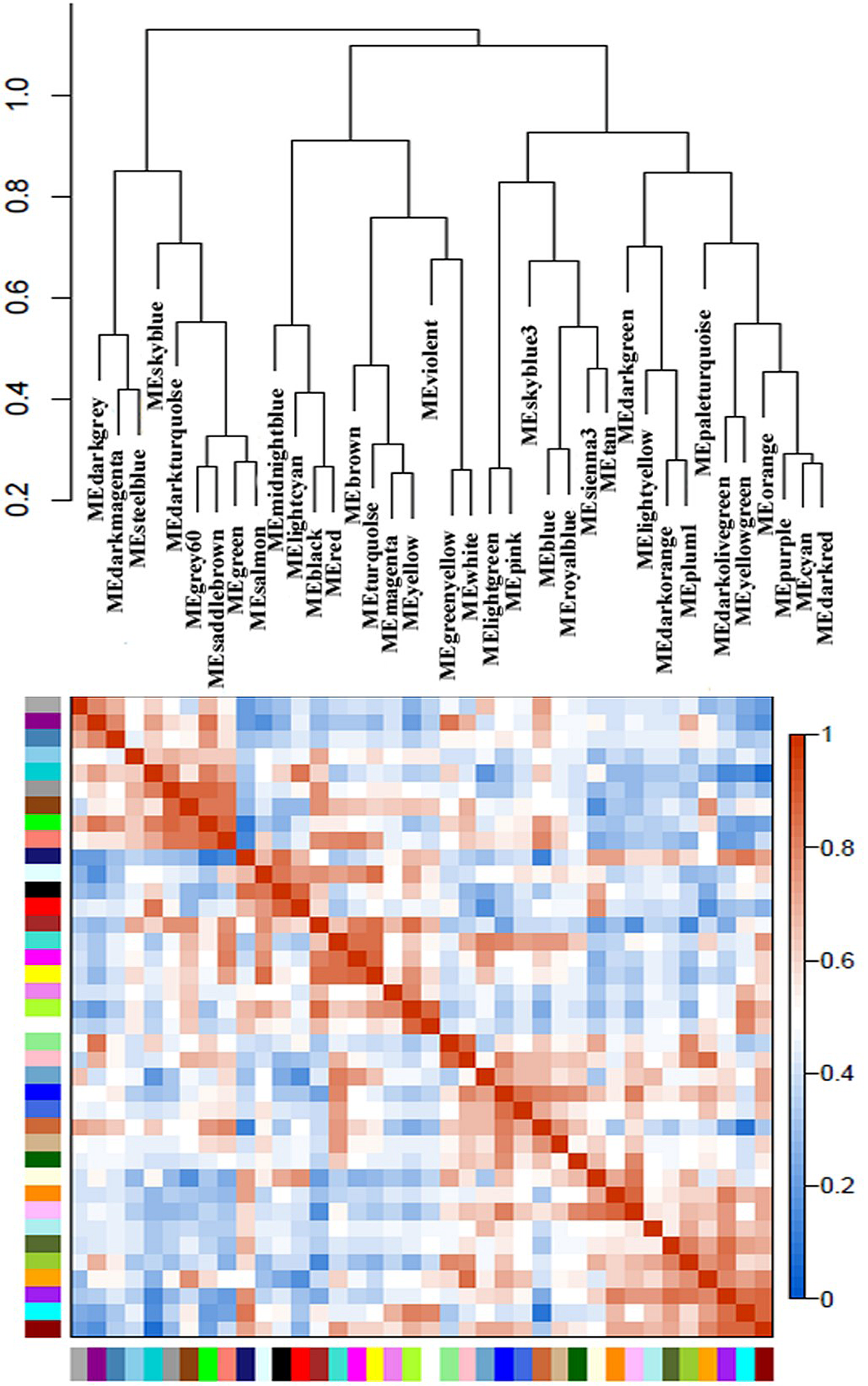


**Supplementary Figure S3.** Gene coexpression modules showing the cluster dendrogram (top) and the heatmap for the correlation coefficient between the modules (bottom).


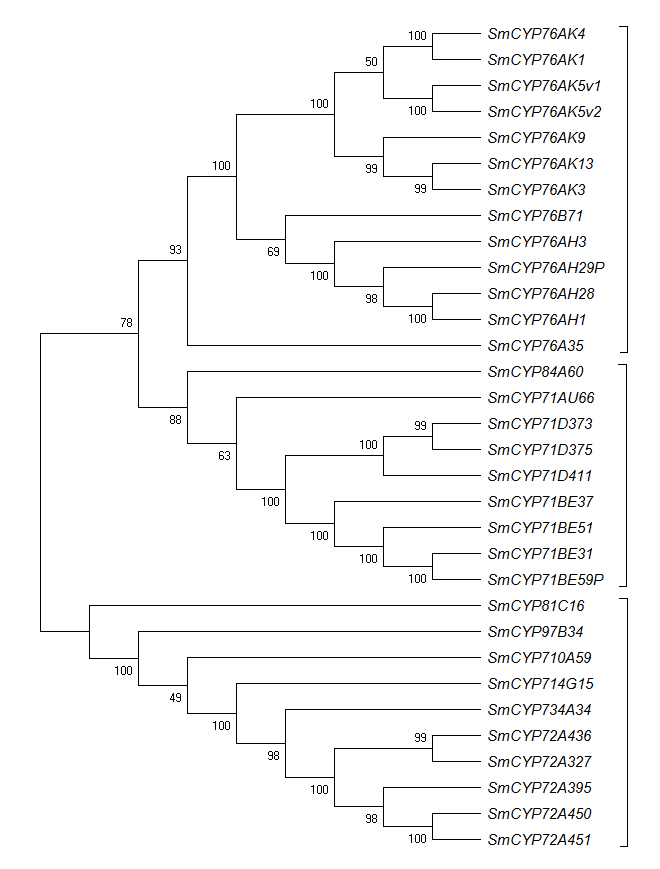


**Supplementary Figure S4.** Phylogenetic relationship of the identified CYPs in “cyan” module.


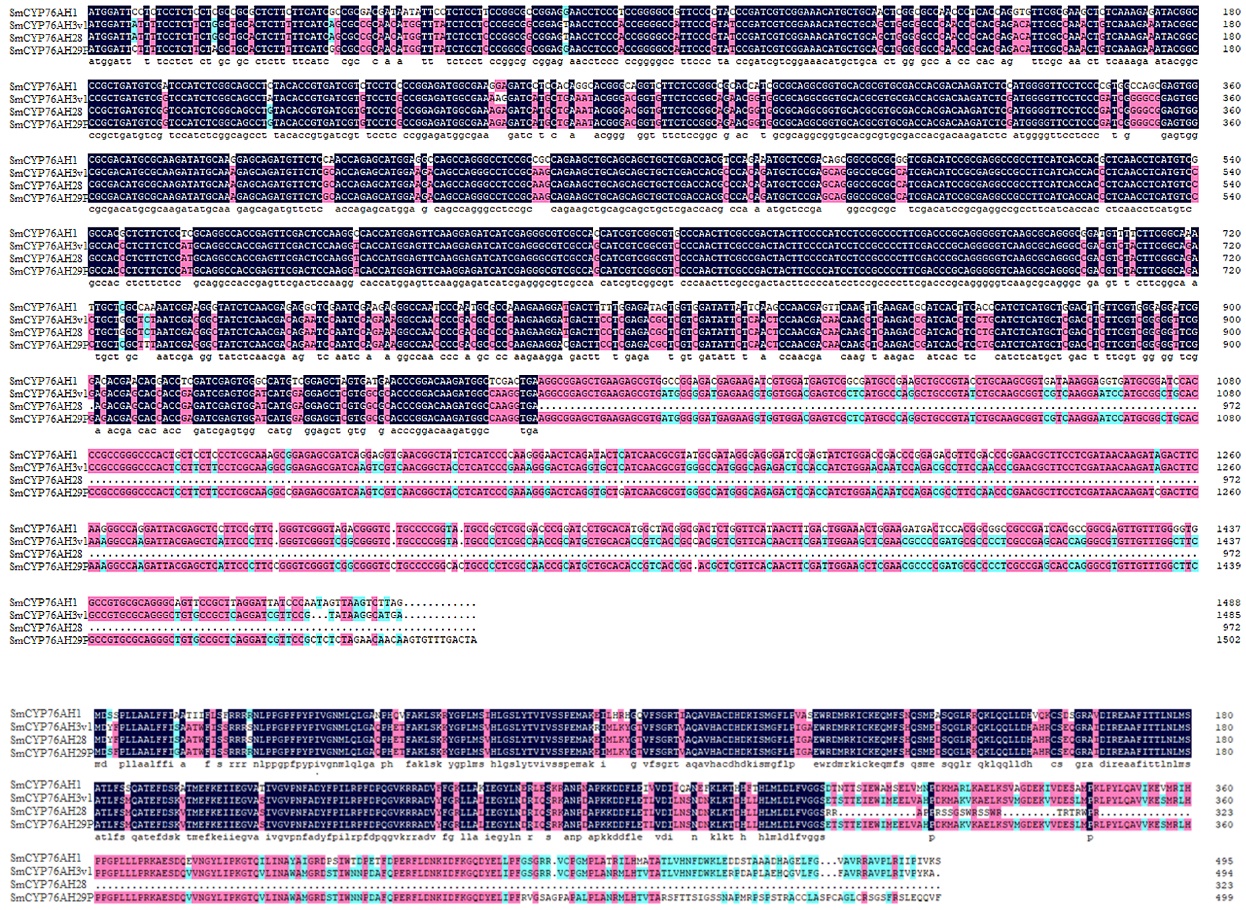


**Supplementary Figure S5.** Sequence alignment of the CYP76AH subfamily in the “cyan” module.


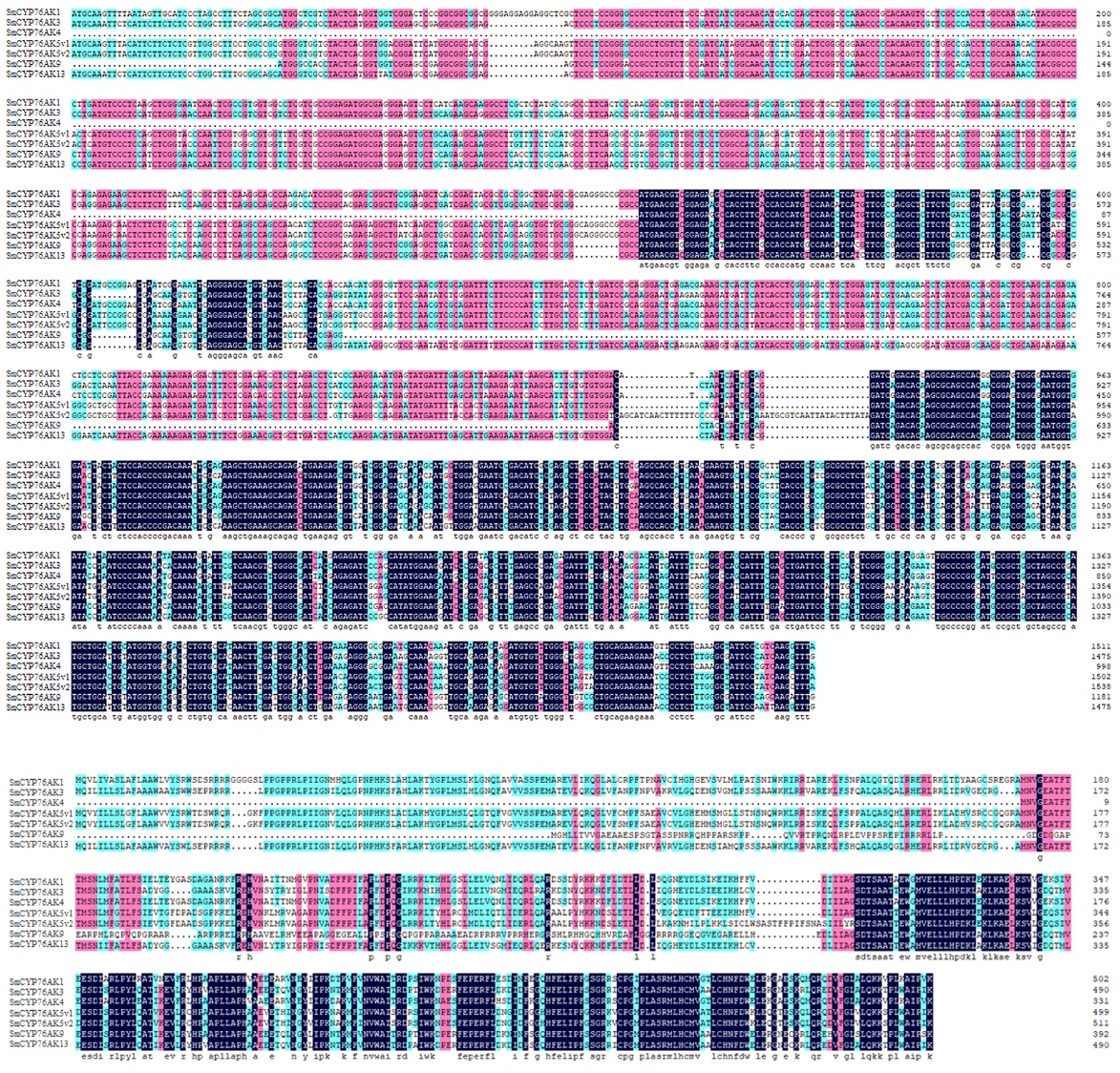


**Supplementary Figure S6.** Sequence alignment of the CYP76AK subfamily in the “cyan” module.


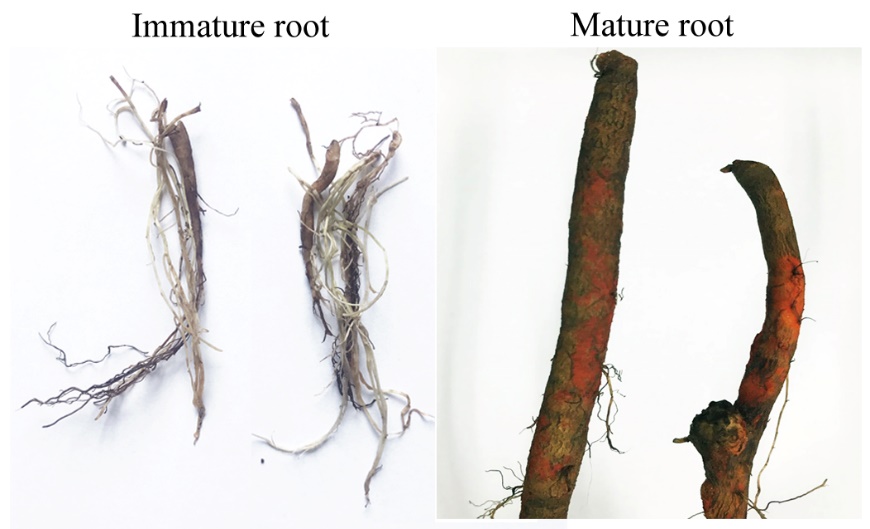


**Supplementary Figure S7.** Immature and mature roots of *S. miltiorrhiza* for transcriptome sequencing.

**Supplementary Table S1.** A statistical summary of clean reads in the transcriptomes of “99-3” and “shh”.

| **Sample ID** | **Raw reads** | **Clean reads** | **Clean bases (G)** | **GC content (%)** | **Q20 (%)** | **Q30 (%)** | **Total mapped** | **Uniquely mapped** |
| --- | --- | --- | --- | --- | --- | --- | --- | --- |
| 99-3R1 | 58,908,808 | 57,709,336 | 8.66 | 48.10 | 98.39 | 95.45 | 46,624,494 (80.79%) | 44440298 (77.01%) |
| 99-3R2 | 52,585,768 | 51,537,084 | 7.74 | 48.01 | 98.45 | 95.57 | 37,638,443 (73.03%) | 36260791 (70.36%) |
| 99-3R3 | 49,057,798 | 48,028,930 | 7.20 | 48.76 | 98.39 | 95.44 | 36,585,926 (76.17%) | 34558503 (71.95%) |
| 99-3RE1 | 51,887,388 | 50,600,996 | 7.60 | 47.89 | 98.30 | 95.33 | 39,194,562 (77.46%) | 37231728 (73.58%) |
| 99-3RE2 | 54,794,632 | 53,498,968 | 8.02 | 48.85 | 98.36 | 95.39 | 41,307,380 (77.21%) | 39143976 (73.17%) |
| 99-3RE3 | 55,674,834 | 54,425,074 | 8.16 | 47.60 | 98.28 | 95.29 | 38,450,729 (70.65%) | 36431790 (66.94%) |
| 99-3W1 | 64,173,806 | 61,881,492 | 9.28 | 47.54 | 98.01 | 95.05 | 37,057,751 (59.89%) | 34537627 (55.81%) |
| 99-3W2 | 46,663,416 | 45,731,024 | 6.86 | 47.61 | 98.41 | 95.47 | 25,444,430 (55.64%) | 24293258 (53.12%) |
| 99-3W3 | 55,674,834 | 54,425,074 | 8.12 | 47.59 | 98.28 | 95.29 | 34,444,430 (63.29%) | 32293258 (59.34%) |
| shhR1 | 49,802,524 | 48,695,652 | 7.30 | 48.23 | 98.33 | 95.36 | 37,202,961 (76.40%) | 35289350 (72.47%) |
| shhR2 | 46,190,694 | 45,244,578 | 6.78 | 48.80 | 98.26 | 95.12 | 31,223,274 (69.01%) | 30591927 (67.61%) |
| shhR3 | 49,325,740 | 48,282,550 | 7.24 | 50.37 | 98.41 | 95.44 | 38,588,002 (79.92%) | 36258298 (75.10%) |
| shhRE1 | 36,400,740 | 35,360,244 | 5.30 | 48.54 | 98.29 | 95.27 | 29,021,822 (82.07%) | 27528506 (77.85%) |
| shhRE2 | 40,487,490 | 39,407,848 | 5.92 | 48.06 | 98.26 | 95.25 | 29,983,540 (76.09%) | 28302991 (71.82%) |
| shhRE3 | 42,248,538 | 41,082,602 | 6.16 | 48.64 | 98.30 | 95.31 | 31,058,769 (75.60%) | 29385422 (71.53%) |
| shhW1 | 50,410,094 | 49,302,984 | 7.40 | 47.26 | 98.38 | 95.44 | 30,889,157 (62.65%) | 29439393 (59.71%) |
| shhW2 | 52,204,846 | 50,911,928 | 7.64 | 46.36 | 98.39 | 95.49 | 28,964,680 (56.89%) | 27609748 (54.23%) |
| shhW3 | 52,486,774 | 50,930,980 | 7.69 | 44.51 | 98.35 | 95.44 | 28,012,537 (55.00%) | 27721718 (53.44%) |

**Supplementary Table S2.** The numerical statistics of the gene expression level in different samples.

| **RPKM Interval** | **0~1** | **1~3** | **3~15** | **15~60** | **>60** |
| --- | --- | --- | --- | --- | --- |
| 99-3R1 | 14356 | 4453 | 8865 | 5471 | 2321 |
| 99-3R2 | 13726 | 4090 | 9044 | 6255 | 2351 |
| 99-3R3 | 13789 | 4214 | 9321 | 6104 | 2038 |
| 99-3RE1 | 14562 | 4596 | 8724 | 5070 | 2514 |
| 99-3RE2 | 14088 | 4123 | 9216 | 5891 | 2148 |
| 99-3RE3 | 14003 | 4146 | 9057 | 5950 | 2310 |
| 99-3W1 | 11606 | 3968 | 9871 | 7702 | 2319 |
| 99-3W2 | 13391 | 3975 | 9072 | 6684 | 2344 |
| 99-3W3 | 12518 | 4186 | 9597 | 6826 | 2339 |
| shhR1 | 15432 | 3968 | 8087 | 5557 | 2422 |
| shhR2 | 15082 | 3914 | 8760 | 5554 | 2156 |
| shhR3 | 15198 | 4029 | 8667 | 5398 | 2174 |
| shhRE1 | 15496 | 3707 | 8289 | 5615 | 2359 |
| shhRE2 | 15082 | 3960 | 8352 | 5647 | 2425 |
| shhRE3 | 15963 | 4017 | 8077 | 5040 | 2369 |
| shhW1 | 13698 | 3921 | 8782 | 6485 | 2580 |
| shhW2 | 13065 | 3873 | 8858 | 7079 | 2591 |
| shhW3 | 12745 | 3834 | 9193 | 7177 | 2517 |

**Supplementary Table S3.** DEGs annotated against biosynthesis of secondary metabolites by KEGG analysis.

| **KEGG** | **Gene_id** | **Gene name** | **KEGG** | **Gene_id** | **Gene name** |
| --- | --- | --- | --- | --- | --- |
| Terpenoid backbone biosynthesis | SMil_00000684-RA_Salv | SmAACT1 | Biosynthesis of secondary metabolites | SMil_00006882-RA_Salv | SmCYP71D441v1 |
|  | SMil_00001042-RA_Salv | SmMDS |  | SMil_00007040-RA_Salv | SmCYP81B82 |
|  | SMil_00001873-RA_Salv | SmMDC |  | SMil_00007725-RA_Salv |  |
|  | SMil_00001874-RA_Salv |  |  | SMil_00008485-RA_Salv |  |
|  | SMil_00003124-RA_Salv | SmPMK |  | SMil_00008642-RA_Salv |  |
|  | SMil_00003204-RA_Salv | SmIDI1 |  | SMil_00009253-RA_Salv |  |
|  | SMil_00004388-RA_Salv | SmHMGS1 |  | SMil_00009920-RA_Salv |  |
|  | SMil_00005164-RA_Salv | SmGGPPS1 |  | SMil_00009979-RA_Salv |  |
|  | SMil_00006065-RA_Salv |  |  | SMil_00009981-RA_Salv |  |
|  | SMil_00009578-RA_Salv | SmHMGR2 |  | SMil_00010118-RA_Salv |  |
|  | SMil_00010132-RA_Salv | SmGPPS.SSUII.1 |  | SMil_00010119-RA_Salv |  |
|  | SMil_00013040-RA_Salv |  |  | SMil_00010521-RA_Salv | SmCYP714G15 |
|  | SMil_00013549-RA_Salv | SmMK |  | SMil_00010536-RA_Salv |  |
|  | SMil_00017330-RA_Salv | SmDXS2 |  | SMil_00010537-RA_Salv |  |
|  | SMil_00018556-RA_Salv |  |  | SMil_00010538-RA_Salv |  |
|  | SMil_00018777-RA_Salv | SmDXR |  | SMil_00010878-RA_Salv |  |
|  | SMil_00019250-RA_Salv | SmHDR1 |  | SMil_00011075-RA_Salv |  |
|  | SMil_00020797-RA_Salv | SmHMGS2 |  | SMil_00011393-RA_Salv |  |
|  | SMil_00021873-RA_Salv | SmGGPPS3 |  | SMil_00011658-RA_Salv |  |
|  | SMil_00027819-RA_Salv | SmCMK |  | SMil_00011733-RA_Salv |  |
|  | SMil_00028353-RA_Salv | SmHDR3 |  | SMil_00012471-RA_Salv |  |
|  | SMil_00030061-RA_Salv |  |  | SMil_00013043-RA_Salv | SmCYP92B37 |
| Diterpenoid biosynthesis | Novel02514 |  |  | SMil_00013368-RA_Salv | SmCYP736A161 |
|  | SMil_00003277-RA_Salv | SmCYP76AK1 |  | SMil_00013370-RA_Salv | SmCYP736A173 |
|  | SMil_00003815-RA_Salv | SmKSL1 |  | SMil_00013642-RA_Salv |  |
|  | SMil_00015959-RA_Salv | SmCPS1 |  | SMil_00014256-RA_Salv |  |
|  | SMil_00019625-RA_Salv | SmCPR2 |  | SMil_00014441-RA_Salv |  |
|  | SMil_00020972-RA_Salv | SmCYP76AH3v2 |  | SMil_00015127-RA_Salv |  |
|  | SMil_00024115-RA_Salv | SmCYP82U11P |  | SMil_00015145-RA_Salv |  |
|  | SMil_00024715-RA_Salv | SmCYP82U5 |  | SMil_00015223-RA_Salv |  |
|  | SMil_00028460-RA_Salv | Sm2OGD-15 |  | SMil_00015949-RA_Salv |  |
|  | SMil_00029757-RA_Salv | SmCYP76AH3v1 |  | SMil_00016451-RA_Salv |  |
|  | SMil_00029991-RA_Salv |  |  | SMil_00017658-RA_Salv | SmCYP81Q69 |
| Biosynthesis of secondary metabolites | SMil_00001864-RA_Salv |  |  | SMil_00017973-RA_Salv |  |
|  | SMil_00003567-RA_Salv |  |  | SMil_00018362-RA_Salv |  |
|  | SMil_00004980-RA_Salv |  |  | SMil_00018425-RA_Salv |  |
|  | SMil_00015129-RA_Salv |  |  | SMil_00018535-RA_Salv |  |
|  | SMil_00016476-RA_Salv |  |  | SMil_00018743-RA_Salv |  |
|  | SMil_00020973-RA_Salv | SmSmCYP76AH1 |  | SMil_00019156-RA_Salv | SmCYP71D486Pv1 |
|  | SMil_00001430-RA_Salv |  |  | SMil_00019458-RA_Salv |  |
|  | SMil_00008028-RA_Salv |  |  | SMil_00019674-RA_Salv | SmCYP71D374 |
|  | SMil_00001923-RA_Salv | SmCYP94A49v1 |  | SMil_00019675-RA_Salv |  |
|  | SMil_00003699-RA_Salv |  |  | SMil_00019689-RA_Salv |  |
|  | SMil_00005770-RA_Salv |  |  | SMil_00019690-RA_Salv |  |
|  | SMil_00008346-RA_Salv |  |  | SMil_00019824-RA_Salv | SmCYP81B76 |
|  | SMil_00012667-RA_Salv | SmCYP92B28 |  | SMil_00020020-RA_Salv |  |
|  | SMil_00013409-RA_Salv |  |  | SMil_00020281-RA_Salv |  |
|  | SMil_00022498-RA_Salv | SmCYP75B79 |  | SMil_00020311-RA_Salv |  |
|  | SMil_00029628-RA_Salv |  |  | SMil_00020829-RA_Salv |  |
|  | SMil_00000124-RA_Salv |  |  | SMil_00021286-RA_Salv |  |
|  | SMil_00002861-RA_Salv |  |  | SMil_00021403-RA_Salv | SmCYP92A125P |
|  | SMil_00004775-RA_Salv |  |  | SMil_00021527-RA_Salv |  |
|  | SMil_00004776-RA_Salv |  |  | SMil_00022184-RA_Salv |  |
|  | SMil_00006253-RA_Salv | SmCYP71AT91 |  | SMil_00022185-RA_Salv |  |
|  | SMil_00006741-RA_Salv |  |  | SMil_00022237-RA_Salv |  |
|  | SMil_00006937-RA_Salv |  |  | SMil_00022456-RA_Salv |  |
|  | SMil_00008529-RA_Salv |  |  | SMil_00022821-RA_Salv |  |
|  | SMil_00009123-RA_Salv |  |  | SMil_00022822-RA_Salv |  |
|  | SMil_00011394-RA_Salv |  |  | SMil_00022912-RA_Salv | SmCYP710A59 |
|  | SMil_00011873-RA_Salv |  |  | SMil_00024176-RA_Salv | SmCYP71D373 |
|  | SMil_00014364-RA_Salv |  |  | SMil_00024215-RA_Salv |  |
|  | SMil_00015535-RA_Salv |  |  | SMil_00024237-RA_Salv |  |
|  | SMil_00019285-RA_Salv |  |  | SMil_00024239-RA_Salv |  |
|  | SMil_00019799-RA_Salv |  |  | SMil_00024363-RA_Salv | SmCYP71D375 |
|  | SMil_00020203-RA_Salv |  |  | SMil_00024737-RA_Salv | SmCYP76A35 |
|  | SMil_00022886-RA_Salv |  |  | SMil_00025219-RA_Salv |  |
|  | SMil_00022945-RA_Salv | SmCYP81Q41 |  | SMil_00025683-RA_Salv |  |
|  | SMil_00022976-RA_Salv |  |  | SMil_00026081-RA_Salv |  |
|  | SMil_00023199-RA_Salv |  |  | SMil_00026324-RA_Salv |  |
|  | SMil_00023200-RA_Salv |  |  | SMil_00026445-RA_Salv |  |
|  | SMil_00023522-RA_Salv |  |  | SMil_00026853-RA_Salv |  |
|  | SMil_00024524-RA_Salv |  |  | SMil_00027251-RA_Salv |  |
|  | SMil_00024925-RA_Salv |  |  | SMil_00027315-RA_Salv |  |
|  | SMil_00025338-RA_Salv |  |  | SMil_00027689-RA_Salv |  |
|  | SMil_00026018-RA_Salv |  |  | SMil_00027827-RA_Salv |  |
|  | SMil_00026135-RA_Salv |  |  | SMil_00027905-RA_Salv |  |
|  | SMil_00026609-RA_Salv | SmCYP98A75 |  | SMil_00027937-RA_Salv |  |
|  | SMil_00028636-RA_Salv | SmCYP98A78 |  | SMil_00028004-RA_Salv | SmCYP71BE31 |
|  | SMil_00028734-RA_Salv |  |  | SMil_00028018-RA_Salv |  |
|  | SMil_00029430-RA_Salv |  |  | SMil_00028244-RA_Salv |  |
|  | SMil_00029919-RA_Salv |  |  | SMil_00028336-RA_Salv |  |
|  | SMil_00030200-RA_Salv | SmCYP98A77 |  | SMil_00028702-RA_Salv |  |
|  | SMil_00030382-RA_Salv |  |  | SMil_00029306-RA_Salv | SmCYP71D410v2 |
|  | Novel00280 |  |  | SMil_00029534-RA_Salv |  |
|  | Novel00823 |  |  | SMil_00030472-RA_Salv |  |
|  | Novel02146 |  |  | SMil_00000716-RA_Salv | SmCYP73A120 |
|  | Novel04116 |  |  | SMil_00001007-RA_Salv |  |
|  | SMil_00000114-RA_Salv |  |  | SMil_00004379-RA_Salv |  |
|  | SMil_00001211-RA_Salv |  |  | SMil_00006292-RA_Salv |  |
|  | SMil_00001212-RA_Salv | SmCYP71D465 |  | SMil_00007576-RA_Salv |  |
|  | SMil_00001220-RA_Salv |  |  | SMil_00010058-RA_Salv |  |
|  | SMil_00001429-RA_Salv |  |  | SMil_00010061-RA_Salv |  |
|  | SMil_00001931-RA_Salv |  |  | SMil_00010062-RA_Salv |  |
|  | SMil_00003273-RA_Salv |  |  | SMil_00010377-RA_Salv |  |
|  | SMil_00003727-RA_Salv | SmCYP71AT103 |  | SMil_00011084-RA_Salv |  |
|  | SMil_00004136-RA_Salv |  |  | SMil_00011824-RA_Salv |  |
|  | SMil_00004552-RA_Salv |  |  | SMil_00012897-RA_Salv |  |
|  | SMil_00004762-RA_Salv |  |  | SMil_00014678-RA_Salv |  |
|  | SMil_00004763-RA_Salv |  |  | SMil_00019884-RA_Salv |  |
|  | SMil_00004765-RA_Salv |  |  | SMil_00019885-RA_Salv |  |
|  | SMil_00004789-RA_Salv | SmCYP71D487P |  | SMil_00022916-RA_Salv |  |
|  | SMil_00005156-RA_Salv |  |  | SMil_00022930-RA_Salv |  |
|  | SMil_00005507-RA_Salv |  |  | SMil_00024875-RA_Salv |  |
|  | SMil_00005680-RA_Salv |  |  | SMil_00024924-RA_Salv |  |
|  | SMil_00005724-RA_Salv |  |  | SMil_00026350-RA_Salv |  |
|  | SMil_00005904-RA_Salv | SmCYP71BE51 |  | SMil_00026871-RA_Salv |  |
|  | SMil_00006129-RA_Salv |  |  | SMil_00027981-RA_Salv |  |
|  | SMil_00006325-RA_Salv | SmSDR-4 |  | SMil_00028637-RA_Salv |  |
|  | SMil_00006403-RA_Salv |  |  | SMil_00028801-RA_Salv |  |
|  | SMil_00006873-RA_Salv |  |  |  |  |

**Supplementary Table S4.** Hub genes in the “cyan” module.

| **Gene ID** | **Edge** | **Description** | **GO & KEGG pathway** |
| --- | --- | --- | --- |
| SMil_00000637-RA_Salv | 400 | \ | \ |
| SMil_00001042-RA_Salv | 400 | *S. miltiorrhiza* 2-C-methyl-D-erythritol 2,4-cyclodiphosphate synthase, **SmMDS** | terpenoid backbone biosynthesis |
| SMil_00004120-RA_Salv | 400 | \ | \ |
| SMil_00007388-RA_Salv | 400 | kinesin-like protein KIN-13B | biological process, binding |
| SMil_00009171-RA_Salv | 400 | putative aldo/keto reductase 1 | \ |
| SMil_00009198-RA_Salv | 400 | \ | \ |
| SMil_00010521-RA_Salv | 400 | *S. miltiorrhiza* cytochrome P450, **SmCYP714G15** | biosynthesis of secondary metabolites |
| SMil_00012646-RA_Salv | 400 | \ | \ |
| SMil_00012949-RA_Salv | 400 | membrane steroid-binding protein 2-like | tetrapyrrole binding |
| SMil_00015420-RA_Salv | 400 | \ | \ |
| SMil_00015551-RA_Salv | 400 | receptor-like protein kinase | protein kinase activity |
| SMil_00016079-RA_Salv | 400 | \ | biological process |
| SMil_00016948-RA_Salv | 400 | BAG-associated GRAM protein 1 | molecular function |
| SMil_00027268-RA_Salv | 400 | pleiotropic drug resistance protein 1-like | catalytic activity |
| SMil_00005164-RA_Salv | 400 | *S. miltiorrhiza* geranylgeranyl diphosphate synthase, **SmGGPPS1** | terpenoid backbone biosynthesis |
| SMil_00005033-RA_Salv | 399 | E3 ubiquitin-protein ligase | transferase activity |
| SMil_00005534-RA_Salv | 399 | serine/threonine-protein kinase | protein kinase activity |
| SMil_00014147-RA_Salv | 399 | \ | \ |
| SMil_00015767-RA_Salv | 399 | \ | \ |
| SMil_00020920-RA_Salv | 399 | sugar phosphate/phosphate translocator | transmembrane transport |
| SMil_00022301-RA_Salv | 399 | GPI-anchored protein | \ |
| SMil_00023166-RA_Salv | 399 | E3 ubiquitin-protein ligase BOI-like | transferase activity |
| SMil_00023279-RA_Salv | 399 | \ | transporter activity |
| SMil_00026252-RA_Salv | 399 | putative receptor-like protein kinase | protein kinase activity |
| SMil_00027184-RA_Salv | 399 | \ | plant hormone signal transduction |
| SMil_00027996-RA_Salv | 399 | \ | \ |
| SMil_00028282-RA_Salv | 399 | \ | \ |
| SMil_00009911-RA_Salv | 398 | fasciclin-like arabinogalactan protein 17 | \ |
| SMil_00026148-RA_Salv | 398 | serine/threonine protein phosphatase 2A | mRNA surveillance pathway |
| SMil_00018866-RA_Salv | 393 | acyl-acyl carrier protein thioesterase ATL3 | \ |
| SMil_00019626-RA_Salv | 392 | serine/threonine-protein kinase | protein kinase activity |
| SMil_00015430-RA_Salv | 391 | wall-associated receptor kinase-like 8 | protein kinase activity |
| SMil_00024737-RA_Salv | 391 | *S. miltiorrhiza* cytochrome P450, **SmCYP76A35** | biosynthesis of secondary metabolites |

**Supplementary Table S5.** Sequence similarity matrix of the CYP76AH and CYP76AK subfamilies in the “cyan” module.

| **CYP subfamily** | **Identified CYPs** | **Amino acid sequence similarity with SmCYP76AH1** | **Amino acid sequence similarity with SmCYP76AH3** | **Amino acid sequence similarity with SmCYP76AK1** |
| --- | --- | --- | --- | --- |
| CYP76AH | **SmCYP76AH1** | 100% | 79.39% | / |
|  | **SmCYP76AH3** | 79.39% | 100% | / |
|  | SmCYP76AH28 | 49.90% | 61.34% | / |
|  | SmCYP76AH29P | 72.8% | 88.80% | / |
| CYP76AK | **SmCYP76AK1** | / | / | 100% |
|  | SmCYP76AK3 | / | / | 69.78% |
|  | SmCYP76AK4 | / | / | 63.62% |
|  | SmCYP76AK5v1 | / | / | 69.18% |
|  | SmCYP76AK5v2 | / | / | 66.02% |
|  | SmCYP76AK9 | / | / | 37.38% |
|  | SmCYP76AK13 | / | / | 69.18% |

**Supplementary Table S6.** Primers used for qRT-PCR.

| **Primer Name** | **Primer Sequence (5’ to 3’)** |
| --- | --- |
| CYP76AH1-F | CCCAACTTCGCCGACTACTT |
| CYP76AH1-R | AGTCATCCTTCTTTGGCGCA |
| CYP76A35-F | AGCATCGATCACAAAGGGCA |
| CYP76A35-R | AGCCCTGTCATCTCCCTCAT |
| CYP76AK9-F | AAGCTCTTCTCCCACCAAGC |
| CYP76AK9-R | GAGTTCACGTGCTCCCTCAA |
| CYP76AK13-F | GCCCGAGCGATTTCTGGATA |
| CYP76AK13-R | TTTTCTTCTGCAGCGCCAAC |
| CYP76B71-F | GCCCCAAGAGATTGACCGAA |
| CYP76B71-R | CTCTCGCACTGTTTCCCCTT |
| CYP710A59-F | GGCCACCTCTTCGATTTCCT |
| CYP710A59-R | CGCCTCCGTGAACTTCATCT |
| CYP71BE59P-F | TCGCTGCGAGAGAAAGTGTT |
| CYP71BE59P-R | AGCAAAGCCTCATGTTCCGT |
| CYP72A436-F | GTTTGAGCTGCGACCAAGTT |
| CYP72A436-R | TGCACATTGAAACTCCATCCA |
| CYP72A327-F | GCCTTCTGTTTGAGCTGTGA |
| CYP72A327-R | TCGGCACATATCTCCATCCA |
| CYP72A450-F | TTACGAGGTGGATGTGTGGC |
| CYP72A450-R | TGGGCACAAATCTCCATCCC |
| CYP72A451-F | CAAGGCACAAAAGGGTCCAG |
| CYP72A451-R | AGTAATCAAACTGAGAGGCGC |
